# Protective Effect of Genistein against Neuronal Degeneration in *ApoE^−/−^* Mice Fed a High-Fat Diet

**DOI:** 10.3390/nu8110692

**Published:** 2016-10-31

**Authors:** Yoon-Jin Park, Je Won Ko, Sookyoung Jeon, Young Hye Kwon

**Affiliations:** 1 Department of Food and Nutition, Seoul National University, Seoul 08826, Korea; younjjn@snu.ac.kr (Y.-J.P.); kgrami@snu.ac.kr (J.W.K.); sookyoung.jeon@gmail.com (S.J.); 2 Research Institute of Human Ecology, Seoul National University, Seoul 08826, Korea

**Keywords:** *ApoE^−/−^* mice, brain, genistein, neurodegeneration, neuroinflammation

## Abstract

Altered cholesterol metabolism is believed to play a causal role in major pathophysiological changes in neurodegeneration. Several studies have demonstrated that the absence of apolipoprotein E (ApoE), a predominant apolipoprotein in the brain, leads to an increased susceptibility to neurodegeneration. Previously, we observed that genistein, a soy isoflavone, significantly alleviated apoptosis and tau hyperphosphorylation in SH-SY5Y cells. Therefore, we investigated the neuroprotective effects of dietary genistein supplementation (0.5 g/kg diet) in the cortex and hippocampus of wild-type C57BL/6 (WT) and *ApoE* knockout (*ApoE^−/−^*) mice fed a high-fat diet (HFD) for 24 weeks. Genistein supplementation alleviated neuroinflammation and peripheral and brain insulin resistance. Reductions in oxidative and endoplasmic reticulum stress were also observed in *ApoE^−/−^* mice fed a genistein-supplemented diet. Beta-secretase 1 and presenilin 1 mRNA levels and beta-amyloid peptide (Aβ) protein levels were reduced in response to genistein supplementation in *ApoE^−/−^* mice but not in WT mice. Although the absence of *ApoE* did not increase tau hyperphosphorylation, genistein supplementation reduced tau hyperphosphorylation in both WT and *ApoE^−/−^* mice. Consistent with this result, we also observed that genistein alleviated activity of c-Jun *N*-terminal kinase and glycogen synthase kinase 3β, which are involved in tau hyperphosphorylation. Taken together, these results demonstrate that genistein alleviated neuroinflammation, Aβ deposition, and hyperphosphorylation in *ApoE^−/−^* mice fed an HFD.

## 1. Introduction

It is increasingly evident that obesity, diabetes mellitus, hypercholesterolemia, and nonalcoholic steatohepatitis (NASH) are associated with multiple aspects of brain pathogenesis [1,2,3]. Importantly, cardiovascular disease risk factors, such as hypercholesterolemia and oxidative stress, are involved in the development of cognitive dysfunction [4,5]. Alzheimer’s disease (AD), the most prevalent neurodegenerative disease in humans, is pathologically characterized by the extracellular deposition of beta-amyloid peptides (Aβ), the formation of intracellular neurofibrillary tangles due to an abnormal hyperphosphorylation of tau at specific epitopes, subsequent neuroinflammation, loss of synaptic plasticity, and neuronal death [6]. As the most cholesterol-rich organ, brain contains about one-fourth of the total body cholesterol for the use of dendritic formation and remodeling, and synaptic plasticity [5]. Epidemiological and experimental studies have shown that dysregulated cholesterol homeostasis may cause AD by regulating trafficking, processing, and clearance of Aβ and its precursor, amyloid precursor protein (APP) [7,8,9,10,11].

Apolipoprotein E (ApoE), the main apolipoprotein released by astrocytes and microglia, transports cholesterol between glial cells and neurons [12]. Although *ApoE^−/−^* mice have been used mostly for cardiovascular disease research [9], the underlying pathology of neurological disorders in *ApoE^−/−^* mice are still inconclusive and contradictory [13]. The induction of oxidative stress and inflammation observed in *ApoE^−/−^* mice may increase the risk of developing neurodegeneration. Some groups have reported that while *ApoE^−/−^* mice develop normally, they begin to show dendritic alterations as early as 4 months of age [14]. Aged *ApoE^−/−^* mice showed significantly impaired cognitive function, which may be caused by a decreased neuronal excitability in hippocampus [15]. However, other studies did not detect learning and behavioral defects in *ApoE^−/−^* mice [16] and rats [17].

Genistein, one of the major isoflavones in soybeans, has antioxidant and phytoestrogenic activities that may contribute to its potential anti-inflammatory, anticarcinogenic, and hypocholesterolemic effects [18,19]. We have previously reported that genistein alleviated NASH as well as hypercholesterolemia and obesity in *ApoE^−/−^* mice fed a high-fat diet (HFD), suggesting that restoration of altered cholesterol metabolism and inhibition of oxidative stress and inflammation may be involved in the protective effect of genistein against NASH development [18]. We also observed the inhibitory effect of isoflavones against endoplasmic reticulum (ER) stress-induced cell death and tau hyperphosphorylation in neuroblastoma cells [20,21]. A neuroprotective effect of genistein in a Parkinson’s disease mouse model [22] and antioxidant effect of genistein in an AD mouse model were also reported [23]. However, a neuroprotective effect of genistein in overnutrition-induced metabolic disease models is not well understood. Therefore, in the present study, we investigated the effect of *ApoE* deficiency and HFD-induced oxidative stress on neuropathology in mice. Furthermore, we investigated the neuroprotective effect of genistein and its underlying mechanism in *ApoE^−/−^* mouse fed an HFD.

## 2. Materials and Methods

### 2.1. Experimental Animals and Diets

Experimental diets and animals were as previously described [18]. Briefly, male wild-type (WT) C57BL/6 mice and *ApoE^−/−^* mice were purchased from Japan SLC., Inc. (Hamamatsu, Japan) at 6 weeks of age, acclimated with chow diet for 1 week, and fed either an HFD or an HFD containing 0.05% genistein (LC Laboratories, Woburn, MA, USA) for 24 weeks. Mice were maintained in a temperature—(22 ± 3 °C) and humidity—(50% ± 10%) controlled room. At the end of the experiments, the mice fasted for 12 h and blood samples were rapidly obtained by cardiac puncture. Brains were rapidly dissected for hippocampus and cortical tissue, snap frozen immediately in liquid nitrogen, and stored at −80 °C until analysis. Animal studies were approved by the Institutional Animal Care and Use Committee of the Seoul National University (SNU-110524-1).

### 2.2. Analyses of Serum

Serum glucose levels were measured using a commercially available kit (Asan Pharmaceutical Co., Seoul, Korea). Serum insulin level was measured using the ELISA kit (Millipore, Billerica, MA, USA). The insulin resistance index was estimated by the homeostasis model assessment of insulin resistance (HOMA-IR) with the following formula: serum glucose × serum insulin/22.5, with serum glucose in mmol/L and serum insulin in mU/L.

### 2.3. Tissue Extract Preparation and Immunoblotting

Frozen cortex and hippocampus samples were homogenized in an ice-cold protein lysis buffer containing 50 mmol/L Hepes-KOH (pH 7.5), 150 mmol/L NaCl, 1 mmol/L EDTA, 2.5 mmol/L EGTA, 1 mmol/L NaF, 10 mmol/L β-glycerophosphate, 0.1 mmol/L Na_3_VO_4_, 1 mmol/L DTT, 0.1% Tween-20, 10% glycerol, 0.2 mmol/L PMSF, and 1% protease inhibitor cocktail (Sigma, St. Louis, MO, USA). After centrifugation for 30 min at 10,000× *g* at 4 °C, the protein content of the supernatant was determined with a protein assay kit. Proteins were resolved by SDS-PAGE and transferred onto an Immobilon-P membrane (Millipore, USA). Following blocking with either 5% nonfat milk or BSA, membranes were probed with specific primary antibodies and subsequently incubated with horseradish peroxidase (HRP)-linked secondary antibodies for chemiluminescent detection. The band intensities were quantified using Quantity One software (Bio-Rad, Hercules, CA, USA). Primary antibodies were obtained as follows: anti-β-actin (Sigma, USA), anti-Aβ (Santa Cruz Biotechnology, Dallas, TX, USA), anti-phospho-glycogen synthase kinase 3β (GSK-3β; Cell Signaling Technology, Danvers, MA, USA), 70-kDa heat shock cognate protein (HSC70; Santa Cruz Biotechnology), anti-heme oxygenase 1 (HO-1; Santa Cruz Biotechnology, USA), anti-insulin receptor substrate 1 (IRS-1; Cell Signaling Technology), anti-phospho-IRS-1 (Cell Signaling Technology), anti-c-Jun N-terminal kinase (JNK; Cell Signaling Technology), anti-phospho-JNK (Cell Signaling Technology), anti-dephosphorylated tau (clone tau-1; Chemicon, Temecula, CA, USA), anti-total tau (clone tau-5; Lab Vision, Fremont, CA, USA), and anti-ubiquitin (Ub; Santa Cruz Biotechnology).

### 2.4. Total RNA Extraction and Semiquantitative RT-PCR

Total RNA of cortex and hippocampus was isolated using RNAiso reagent (Takara Bio, Shiga, Japan), and cDNA was synthesized using 2 µg of total RNA with the Superscript^®^II Reverse Transcriptase (Invitrogen, Carlsbad, CA, USA). For amplification of cDNA, primers for X-box-protein-1 (Xbp-1; forward 5′-AAACAGAGTAGCAGCTCAGACTGC-3′, reverse 5′-TCCTTCTGGGTAGACCTCTGGGAG-3′) were used. Expression of β-actin was examined as an internal control (forward 5′-TGACCCAGATCATGTTTGAGACC-3′, reverse 5′-CCATACCCAAGAAGGAAGGC-3′). Amplified products of Xbp-1 were further digested by *PstI* to check whether a *PstI* restriction site was lost after inositol-requiring enzyme 1-mediated splicing of mRNA. The amplified products were separated on an agarose gel and visualized with ethidium bromide staining under UV illumination. The bands were quantified with Quantity One software (Bio-Rad, USA).

### 2.5. Quantitative RT-PCR (qRT-PCR) Analysis

Amplification reactions were performed according to the manufacturer’s protocol of the SYBR^®^ Green PCR Master Mix (Applied Biosystems, Foster City, CA, USA). PCR products were verified by melting curve analysis. Mouse ribosomal protein L19 (RPL19) was used as a reference gene to normalize for differences in the amount of total RNA in each sample. Relative gene expression levels were analyzed using the 2^−ΔΔ*C*t^ method. The primer sequences are described in Table 1.

### 2.6. Statistical Analysis

Data were analyzed using SPSS software (ver. 21.0, SPSS Inc., Armonk, NY, USA). For all experiments, one-way ANOVA followed by Duncan’s multiple range test were used to assess statistical significance. Data were expressed as the, mean ± standard error of the mean (SEM), and differences were considered statistically significant at *p* < 0.05.

## 3. Results

### 3.1. Effect of Genistein on Brain Weight in ApoE^−/−^ Mice Fed an HFD

**Figure 1:**
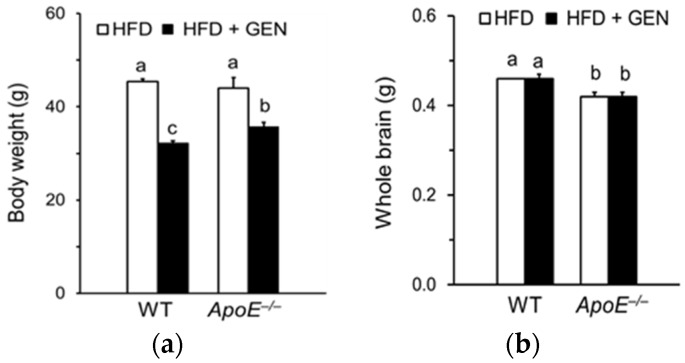
Effects of genistein on (**a**) body weight and (**b**) brain weight of wild-type (WT) and apolipoprotein E knockout (*ApoE^−/−^*) mice fed a high-fat diet (HFD). Each bar represents the mean ± standard error of the mean (SEM) (*n* = 9, 10) and bars with different superscripts are significantly different at *p* < 0.05.

Body weight was significantly reduced by genistein supplementation in both WT and *ApoE^−/−^* mice (Figure 1a). Brain weight of *ApoE^−/−^* mice was significantly lower than that of WT mice (Figure 1b). However, we did not observe the effect of genistein supplementation on brain weight of mice.

### 3.2. Effect of Genistein on Peripheral and Central Insulin Resistance in ApoE^−/−^ Mice Fed an HFD

**Figure 2:**
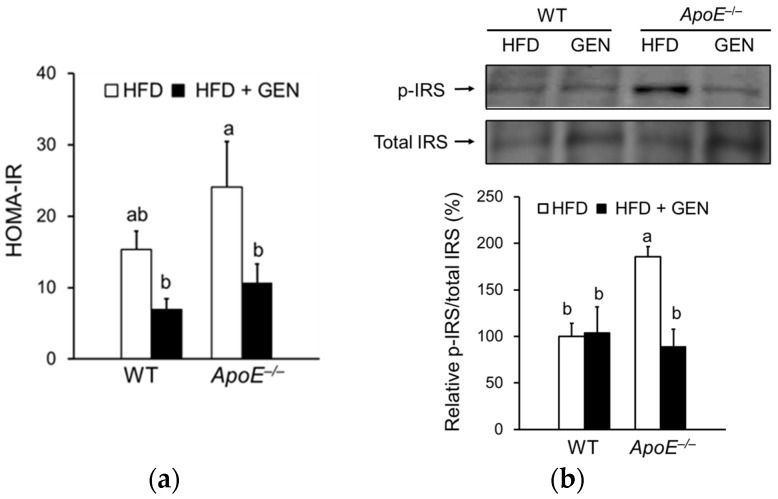
Effects of genistein on insulin resistance of WT and *ApoE^−/−^* mice fed an HFD. (**a**) Homeostasis model assessment of insulin resistance (HOMA-IR) was calculated based on serum glucose and insulin levels (*n* = 5–7); (**b**) relative protein levels of phosphorylated insulin receptor substrate (p-IRS) in the cortex and hippocampus were analyzed by immunoblotting and were normalized to total IRS (*n* = 3). Each bar represents the mean ± SEM and bars with different superscripts are significantly different at *p* < 0.05.

We investigated the effects of genistein supplementation on peripheral and central insulin resistance in WT mice and *ApoE^−/−^* mice. Based on HOMA-IR, genistein supplementation significantly lessened systemic insulin resistance in *ApoE^−/−^* mice (Figure 2a). We also determined insulin resistance in the brain by quantifying phosphorylated form of IRS protein and observed a significant decrease in p-IRS protein levels with genistein supplementation in *ApoE^−/−^* mice (Figure 2b).

### 3.3. Effect of Genistein on ER Stress in the Brain of ApoE^−/−^ Mice Fed an HFD

**Figure 3:**
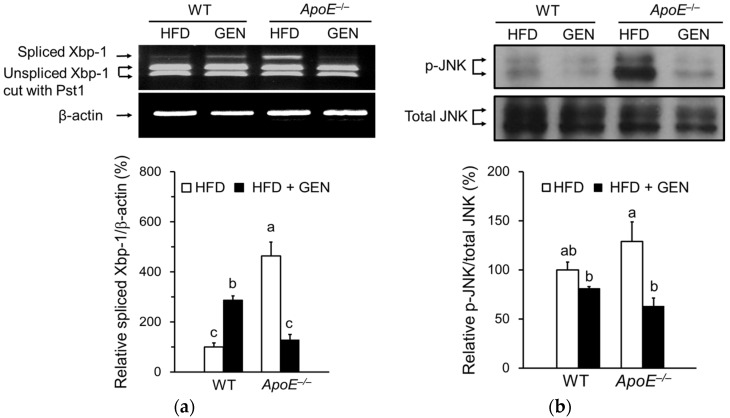
Effects of genistein on endoplasmic reticulum (ER) stress in the cortex and hippocampus of WT and *ApoE^−/−^* mice fed an HFD. (**a**) Relative mRNA expressions of X-box-protein-1 (Xbp-1) spliced form were analyzed by semiquantitative PCR and normalized to beta-actin (*n* = 3); (**b**) relative protein levels of phosphorylated c-Jun N-terminal kinase (p-JNK) were analyzed by immunoblotting and were normalized to total JNK (*n* = 3). Each bar represents the mean ± SEM and bars with different superscripts are significantly different at *p* < 0.05.

ER stress is involved in neuronal toxicity as well as insulin resistance in the brain. We measured mRNA levels of the ER stress-responsive gene, a spliced form of Xbp-1, by semiquantitative RT-PCR (Figure 3a). The activation of unfolded protein response (UPR) involves the concerted action of three proximal ER transmembrane proteins, including inositol-requiring enzyme 1 (IRE1). IRE1 cleaves the Xbp-1 mRNA to remove a small intron, resulting in a translational frameshift that yields a more potent transcriptional activator of UPR-inducible genes [24]. *ApoE^−/−^* mice on HFD significantly increased the spliced form of Xbp-1 mRNA expression compared to WT mice on HFD. We measured the ER stress-mediated activation of JNK in the brain to investigate the mechanism involved in insulin resistance (Figure 3b). Genistein supplementation significantly reduced the active form of JNK, which phosphorylates IRS-1 serine 307, in *ApoE^−/−^* mice.

### 3.4. Effect of Genistein on Oxidative Stress and Inflammation in the Brain of ApoE^−/−^ Mice Fed an HFD

**Figure 4:**
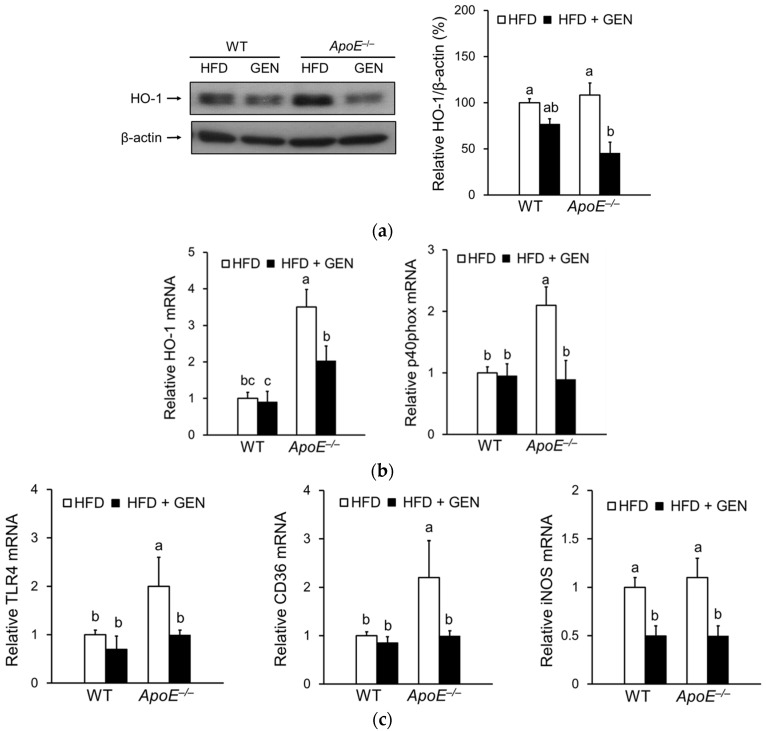
Effects of genistein on oxidative stress and inflammation in the cortex and hippocampus of WT and *ApoE^−/−^* mice fed an HFD. (**a**) Relative protein levels of HO-1 were analyzed by immunoblotting and were normalized to beta-actin (*n* = 3); (**b**) relative mRNA expressions of genes involved in oxidative stress, HO-1 and p40phox, were analyzed by real-time PCR and normalized to ribosomal protein L19 (RPL19) (*n* = 4–5); (**c**) relative mRNA expressions of genes involved in inflammation, TLR4, CD36, and, iNOS were analyzed by real-time PCR and normalized to RPL19 (*n* = 4–5). Each bar represents the mean ± SEM and bars with different superscripts are significantly different at *p* < 0.05.

Previous studies show that oxidative stress is involved in the occurrence of Aβ-induced neurotoxicity [25] and, thereby, in a number of neurodegenerative diseases, including AD [26]. Genistein supplementation significantly reduced HO-1 protein levels in only *ApoE^−/−^* mice, indicating a decrease in oxidative stress (Figure 4a). The mRNA levels of HO-1 and p40phox, a member of nicotinamide adenine dinucleotide phosphate (NADPH) oxidase, were significantly reduced by genistein in *ApoE^−/−^* mice (Figure 4b). NADPH oxidase activation has been suggested to play a major role in the generation of reactive oxygen species in *ApoE^−/−^* mice [27].

In addition to oxidative stress, increasing evidence suggests that neuroinflammation is associated with Aβ-induced learning and memory impairment [28]. As shown in Figure 4c, HFD-fed *ApoE^−/−^* mice showed significantly higher mRNA levels of receptors involved in the amyloid cascade, such as Toll-like receptor (TLR)-4 and CD36, and mRNA levels of inducible nitric oxide synthase (iNOS) compared to WT mice. Genistein supplementation significantly reduced expressions of proinflammatory genes.

### 3.5. Effect of Genistein on Oxidative Stress and Inflammation in the Brain of ApoE^−/−^ Mice Fed an HFD

**Figure 5:**
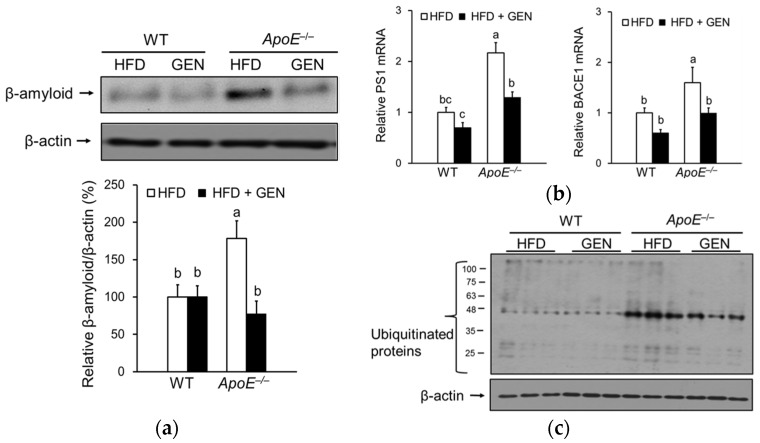
Effects of genistein on beta-amyloid peptide (Aβ) deposition in the cortex and hippocampus of WT and *ApoE^−/−^* mice fed an HFD. (**a**) Relative protein levels of Aβ were analyzed by immunoblotting and were normalized to beta-actin (*n* = 3); (**b**) relative mRNA expressions of genes involved in amyloid precursor protein (APP) processing, BACE1 and PS1, were analyzed by real-time PCR and normalized to RPL19 (*n* = 4–5); (**c**) ubiquitinated protein levels were analyzed by immunoblotting and were normalized to beta-actin (*n* = 3). Each bar represents the mean ± SEM and bars with different superscripts are significantly different at *p* < 0.05.

We measured Aβ protein deposition, a major pathological hallmark of AD, in the cortex and hippocampus. Deficiency of *ApoE* significantly increased Aβ protein levels, which were significantly decreased by genistein supplementation (Figure 5a). Aβ is derived from APP through sequential cleavage by β-secretase (BACE) and γ-secretase. Because the expression and activity of BACE and γ-secretase have been shown to be elevated in the brain of late-onset sporadic AD patients [29], we determined whether genistein regulates mRNA levels of BACE1 and presenilin 1 (PS1), the catalytic subunit of γ-secretase (Figure 5b). Both BACE1 and PS1 mRNA levels were significantly higher in *ApoE^−/−^* mice fed an HFD than WT mice fed an HFD, and were significantly reduced by genistein only in *ApoE^−/−^* mice. Since the impaired ubiquitin-proteasome system in AD could result in an abnormal accumulation of Aβ [30], we determined levels of ubiquitinated proteins and observed that *ApoE* deficiency increased ubiquitinated protein levels (Figure 5c). Genistein supplementation significantly reduced ubiquitinated protein levels in *ApoE^−/−^* mice.

### 3.6. Effect of Genistein on Tau Hyperphosphorylation in the Brain of ApoE^−/−^ Mice Fed an HFD

**Figure 6:**
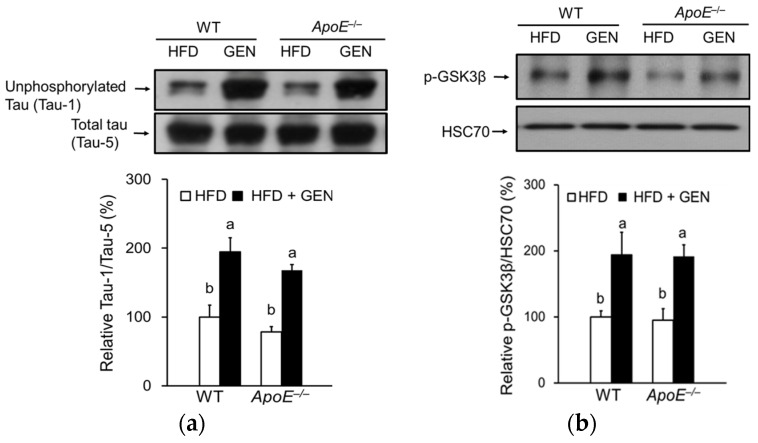
Effects of genistein on tau hyperphosphorylation in the cortex and hippocampus of WT and *ApoE^−/−^* mice fed an HFD. (**a**) Relative protein levels of unphosphorylated tau were analyzed by immunoblotting and were normalized to total tau (*n* = 3); (**b**) relative protein levels of phosphorylated glycogen synthase kinase 3β (GSK-3β) were analyzed by immunoblotting and were normalized to heat shock cognate protein (HSC) 70 (*n* = 3). Each bar represents the mean ± SEM, and bars with different superscripts are significantly different at *p* < 0.05.

Our previous in vitro study reported that genistein and daidzein significantly decreased tau hyperphosphorylation [21]. Tau, which is phosphorylated at over 30 serine/threonine residues in the AD brain [31,32], is a substrate for several protein kinases, including GSK-3β and JNK. GSK-3β is identical to the tau protein kinase I and its activation has been strongly implicated in the abnormal hyperphosphorylation of tau [33,34]. A deficiency of *ApoE* did not significantly change tau phosphorylation in the cortex and hippocampus. Phosphorylation of tau was significantly alleviated by genistein (Figure 6a). Since tau is phosphorylated by GSK-3β, we measured the activation of GSK-3β by immunoblotting and observed a corresponding increase in the inactive, phosphorylated form of GSK-3β in mice fed a genistein-supplemented diet (Figure 6b).

## 4. Discussion

Isoflavones, such as genistein, have antioxidant and neuroprotective effects against chemically induced AD [23,35,36] and diabetes models [37]. However, the effects of genistein on diet-induced AD pathology have not been determined. In this study, we showed that genistein supplementation significantly reduced Aβ accumulation and tau hyperphosphorylation, two main characteristics associated with AD in *ApoE^−/−^* mice fed an HFD. These neuroprotective effects of genistein were associated with inhibition of ER stress- and oxidative stress-mediated insulin resistance and neuroinflammation.

In the present study, hypercholesterolemic *ApoE^−/−^* mice on an HFD did exhibit a larger increase in neurodegeneration compared to WT mice on the same diet. *ApoE* deficiency significantly induced oxidative stress and inflammation in the hippocampus and cortex based on mRNA levels of related genes. Our previous analyses demonstrated that serum cholesterol levels are significantly correlated with systemic inflammation [18], which has a significant effect on the induction of neurodegeneration [4,38]. Therefore, in addition to increased expression of cytokines in the brain per se, systemic inflammation observed in hypercholesterolemic mice would aggravate the neuroinflammation in the brain.

Previous studies have shown that hypercholesterolemia led to APP processing in low-density lipoprotein receptor-deficient mice [10] and in Aβ-injected *ApoE^−/−^* mice with an HFD [8]. Increased APP processing in response to hypercholesterolemia was also observed in the transgenic mice modeling AD on a high-cholesterol diet [39,40]. Consistently, we observed that parameters involved in APP processing, such as BACE1 and PS1 mRNA levels and Aβ protein levels, were significantly higher in *ApoE^−/−^* mice fed an HFD than WT mice fed an HFD. Serum cholesterol levels were significantly associated with Aβ protein levels (*r* = 0.665, *p* = 0.026). In contrast, *ApoE* deficiency did not significantly increase tau hyperphosphorylation and GSK-3β activity and there was no significant correlation between serum cholesterol levels and tau hyperphosphorylation, implying that factor(s) other than hypercholesterolemia may be involved in tau hyperphosphorylation in this model. 

We also observed that *ApoE* deficiency induced insulin resistance in the hippocampus and cortex of mice based on levels of p-IRS, which is phosphorylated by JNK in response to ER stress. Supplementation of genistein significantly reduced the ER stress-mediated induction of insulin resistance. Insulin receptors are present in the hippocampus and cerebral cortex, suggesting a role of insulin in cognitive function [41]. Indeed, insulin resistance in the brain has been shown to influence several aspects of AD pathology, including tau hyperphosphorylation and Aβ processing [42]. Mice with insulin deficiency induced by streptozotocin showed rapid tau hyperphosphorylation, similar to that observed in early AD [43]. Diet-induced insulin resistance also accelerated Aβ pathology by activating γ-secretase and inactivating insulin-degrading enzyme in the Tg2576 AD mouse model [42]. Furthermore, insulin resistance-mediated activation of neurotransmitter catabolizing enzymes, including acetylcholine esterase and monoamine oxidase, was suggested to act as a relative risk factor for brain dysfunction and damage in rats with nonalcoholic fatty liver disease [44]. ER stress has been shown to play an important role in tau phosphorylation. According to a recent study, ER stress may further aggravate APP processing by activating the proteasomal degradation pathway [45].

In addition to cholesterol-lowering and antioxidant effects observed in our previous study [18], genistein has been shown to directly affect brain function by estrogen receptor-mediated processes [46]. The soy-derived isoflavones, mainly genistein and daidzein, have a structure similar to that of 17β-estradiol (E_2_) and are capable of binding to estrogen receptor α and β, which are shown to be expressed in different areas of brain [47]. Genistein and its metabolites were detected in the brain following oral exposure, although their concentrations in the brain were lower than those in reproductive organs, such as prostate, ovary, and uterus [48]. Genistein concentrations in serum and brain of ovariectomized rats after oral administration of genistein at 30 mg/kg body weight for 6 weeks were 190.1 nmol/L and 74.3 ng/g, respectively [49]. Although we did not determine serum total genistein concentration in the present study, it would reach about 0.39–3.36 μmol/L based on a previous study in mice fed an AIN-93G diet supplemented with genistein at various doses (0.0125%–0.1%) for 24 weeks [50]. These reported concentrations are comparable with those in women who consumed soy products [51]. Therefore, further analysis using an animal model treated with the estrogen receptor antagonist would be needed to investigate the role of estrogen receptor- and non-estrogen receptor-mediated mechanisms in neuroprotective effects of genistein.

## 5. Conclusions

We observed significant inhibition of oxidative stress and neuroinflammation in *ApoE^−/−^* mice by genistein supplementation. Genistein supplementation also effectively reduced Aβ formation by reducing gene expression of APP-processing enzymes, including BACE1 and PS1, and reduced tau hyperphosphorylation by inactivating GSK-3β and JNK in the hippocampus and cortex. Furthermore, the present study provides evidence that dysregulated cholesterol metabolism may lead to an accumulation of Aβ and increases in oxidative stress and neuroinflammation.

## Figures and Tables

**Table 1:** Primer sequence for quantitative RT-PCR (qRT-PCR).

Gene	Forward	Reverse
BACE1	GCATGATCATTGGTGGTATC	CCATCTTGAGATCTTGACCA
CD36	TCCTCTGACATTTGCAGGTCTATC	AAAGGCATTGGCTGGAAGAA
HO-1	CCTCACTGGCAGGAAATCATC	CCTCGTGGAGACGCTTTACATA
iNOS	CAGGAGGAGAGAGATCCGATTTA	GCATTAGCATGGAAGCAAAGA
p40phox	CCTGCCCACATTGCCAGCCA	AGACCGGCAGGCTCAGGAGG
PS1	TGCGGCCATCATGATCAGTGTC	ATAAGCCAGGCGTGGATGAC
TLR4	AGGAAGTTTCTCTGGACTAACAAGTTTAGA	AAATTGTGAGCCACATTGAGTTTC

BACE1, β-Secretase 1; CD36, Cluster of differentiation 36; HO-1, Heme oxygenase 1; iNOS, Inducible nitric oxide synthase; p40phox, Neutrophil cytosolic factor 4; PS1, Presenilin-1; TLR4, Toll-like receptor 4.
